# Molecular docking analysis of p53 with Toll-like receptors

**DOI:** 10.6026/97320630017784

**Published:** 2021-09-30

**Authors:** Mohammad Jahoor Alam, Fevzi Bardakci, Sadia Anjum, Shumayla Rasheed Mir, Irfan Ahmad, Mohd Saeed

**Affiliations:** 1 Department of Biology, College of Science, University of Hail, Kingdom of Saudi Arabia; 2 College of Computer Science and Engineering, University of Hail, Kingdom of Saudi Arabia,; 3 Department of Clinical Laboratory Science, College of Applied Medical Science, King Khalid University, Kingdom of Saudi Arabia;

**Keywords:** p53, Toll-like receptor, Interaction, Communication, Docking

## Abstract

P53 is one of the most important proteins for its role in cellular signal transduction pathways. It regulates a wide variety of cellular processes, which includes apoptosis, senescence, cell cycle arrest, differentiation, and DNA repair and replication and cancer dynamics. It is a transcription factor for various cellular proteins. Recent report suggests that P53 is linked with transduction proteins involved in cellular immunity. Toll like receptors are needed for communication in cellular immunity. The interaction between p53 and toll like receptors is reported in various studies. Therefore, it is of interest to document the molecular docking analysis of p53 with Toll-like receptors for further consideration in therapeutic development. In the present paper we studied molecular interaction between p53 and toll like receptors using molecular docking approach. We used open-source tools for molecular docking and analyzing the data. Our molecular docking results suggest there is a promising interaction between p53 and toll like receptors. Our study will be a very useful for molecular therapeutics and drug design strategies. Further, molecular dynamics studies can be useful to determine of the stability of complex form by p53 and toll like receptors.

## Background:

P53 protein act as transcription factor for various cellular proteins such as MDM2, p21, Fas, Bax, p48, PTEN, B99, PAI, related to apoptosis, cell-cycle arrest, senescence, glycolysis, TCA cycle, differentiation, cell fate and suppression of cancer cells progression [1]. The concentration of p53 protein in cells also varies due to its response to a variety of cellular stress, such as, hypoxia, nucleotide depletion, nitric oxide, DNA damage [2]. Recently, role of p53 in cellular immunity have been reported [3]. It is evident that a highly activated form of the p53 protein leads to suppression of inflammation. It is suggested that it activates Toll-like receptors (TLRs) proteins [4]. Cellular immunity acts as defense systems against bacterial, viral and fungal infections [5-7]. Moreover, cells have specialized receptor proteins (such as PRRs (pattern recognition receptors)), which are responsible for recognizing antigens such as bacteria, virus, fungus etc, and activating the proteins which are directly and indirectly associated with cellular immunity. Further, PRRs are specialized in functions due to its recognition of repeating patterns of molecular structures of pathogen, known as PAMP (pathogen associated molecular pattern). TLRs belong to the family of pattern recognition receptors (PRRs) [6,8, 9]. TLRs also participate in signaling of various metabolic pathways linked to apoptosis and suppression of cancer progression [10]. However, the interaction between TLRs and p53 is known [11] with molecular interaction data [12]. Therefore, it is of interest to document the molecular docking analysis of p53 with Toll-like receptors for further consideration in therapeutic development.

## Materials & Methods:

P53 structure data (PDB ID: 2OCJ) was downloaded from RCSB [https://www.rcsb.org/]. We used discovery studio suit for the removal of unnecessary molecules from PDB structure file. Similarly, we also downloaded PDB structure for all human TLRs (from TLR 1 to TLR 10) [PDB ID: 1FYV (TLR1), 1FYW (TLR2), 2MK9 (TLR3), 2Z63 (TLR4), 3JOA (TLR5), 4OM7 (TLR6), 6LVY (TLR7), 5W3M (TLR8), 5Y3M (TLR9), 2J67 (TLR10)]. Protein-protein molecular docking completed using HADDOCK 2.4 (an open-source tools for molecular docking) available at [https://wenmr.science.uu.nl/haddock2.4/] [13]. The results obtained from HADDOCK were further analyzed using PRODIGY (an online tool) [https://wenmr.science.uu.nl/prodigy/] [14] for finding binding affinity as well as dissociation constant of the protein-protein complex formed by p53 and TLRs as shown in Table 1(see PDF). Moreover, we also collected docking parameters such as HADDOCK score, RMSD from the overall lowest-energy structure, Van der Waals energy, Electrostatic energy, Desolvation energy and Z-Score form HADDOCK tools after completion of docking of each TLR receptor with p53 as shown in Table 2(see PDF). Simultaneously, we also performed molecular docking using PyDOCK (an online server) [https://life.bsc.es/pid/pydock/] [15] to further verify the molecular docking interaction between p53 and TLRs. We further finding the stability of p53 and TLRs protein complex using online available statistical split server (SPSERVER) [http://aleph.upf.edu/spserver/] [16]. The output of the SPSERVER has been tabled in Table 3(see PDF). Various statistical parameters were used such as Pair, Ecomb, Es3dc, Elocal, E3dc, E3d, Zpair, Zecomb, Zes3dc, Zelocal and Ze3dc, to find out the stable complex forming. We also used RStudio statistical package to pot heatmap for the protein-protein interaction between p53 and TLRs.

## Results:

We have shown p53 and TLRs interaction obtained from HADDOCK docking in Figure 1(see PDF). It is noticed that that all the TLR protein interact with p53 protein. We observed hydrogen as well as electrostatic bond near the interface between p53 and each TRL as shown in Figure 1(see PDF). It is found that among all interaction between protein-protein, TLR 4 interaction is stable as shown Table 1 and Table 2 (see PDF). The binding affinity between TLR4 and p53 was found to be -18.8, which is comparatively higher among all interactions. Further, dissociation constant is also comparatively higher for p53 and TLR4 interaction. The higher is the stability constant (dissociation constant), higher is the stability of the complex. Molecular docking results using PyDOCK (online server for protein-protein docking) as shown in Figure 2(see PDF). It is noticed from results shown in Figure 2(see PDF) that there is comparatively strong interaction between p53-TLR4, p53-TLR7, p53-TLR8 and p53-TLR9.

We assessed the stability of the complex formation between p53 and various TLRs proteins using SPSERVER statistical tools as shown in Figure 3(see PDF). We observed that all TLR proteins forms stable complex with p53. Moreover, it is also noticed that from results shown in Figure 3(see PDF) that there is comparatively strong stability between p53-TLR4, p53-TLR7, p53-TLR8 and p53-TLR9 complexes. Heatmap plot, as shown in Figure 4(see PDF), further confirmed the interaction between p53 and TLRs are very prominent. Moreover, it is again noticed that there is comparatively strong stability between p53-TLR4, p53-TLR7, p53-TLR8 and p53-TLR9 complexes.

## Discussion:

The role of p53 in apoptosis and cancer is reported in various research literatures [1,2,17,20,21]. Moreover, the role of p53 in cellular immunity is still a challenging area of the research [3,4,22]. Similarly, the role of TLRs is very much studied in cellular immunity [23-27]. However, role of TLRs in apoptosis and cancer is still few reported [10,28-31]. Docking result suggests that there are strong possibilities of interaction between p53 and TLRs proteins. The HADDOCK docking results in Figure 1(see PDF), suggests that TLR1 to TLR10 proteins interacts with p53 protein. Again, the binding affinity and dissociation constant as shown in Table 1(see PDF) further support the docking results obtained from HADDOCK. The PRODIGY analysis suggests that comparatively strong interaction between p53-TLR4, p53-TLR7, p53-TLR8 and p53-TLR9 complexes. Moreover, PyDock server docking results further suggest that there are interactions between p53 and TLRs proteins as shown in Figure 2(see PDF). The statistical assessment of the stability of complexs between p53 and various TLRs proteins also suggests and support the docking results as shown in Figure 3(see PDF). Moreover, statistical analysis results also suggest that there is comparatively strong stability between p53-TLR4, p53-TLR7, p53-TLR8 and p53-TLR9 complexes. Finally, Heatmap plot as shown in Figure 4(see PDF), clarify the interactions between p53 and TLRs proteins. Heatmap plot also suggests that there is comparatively strong stable complex between p53-TLR4, p53-TLR7, p53-TLR8 and p53-TLR9.

## Conclusions:

We document the Molecular docking analysis of p53 with Toll-like receptors. Moreover, the interaction between p53-TLR4, p53-TLR7, p53-TLR8 and p53-TLR9 is found to be stable for the understanding of their molecular mechanism. Our study will be a very useful for molecular therapeutics and drug design studies for cellular immunity and cancer. In future path, for more clarity, molecular dynamics studies can be useful to determine of the stability of complex form by p53 and toll like receptors.
